# Diffuse Large B Cell Lymphoma with Cutaneous and Gastrointestinal Involvement

**DOI:** 10.1155/2022/2687291

**Published:** 2022-02-27

**Authors:** Aye-Mya-Mya Kyaw, Than-Than Aye, Lin-Lin Htun

**Affiliations:** ^1^Gastroenterology Department, Thingangyun General Hospital, Yangon, Myanmar; ^2^Pathology Department, Thingangyun General Hospital, Yangon, Myanmar

## Abstract

Diffuse large B cell lymphoma (DLBCL) is the histological subtype of non-Hodgkin's lymphoma, representing approximately 30%. The most common primary extranodal sites of DLBCL are the gastrointestinal (GI) tract, the head and neck, and the skin/soft tissue. We report a case of DLBCL with cutaneous involvement presenting with skin nodules and GI manifestations such as obstructive jaundice and upper GI bleeding. Malignant cystic pancreatic tumor occupying the head and body with invasion to lower end of common bile duct and periampullary region causing biliary obstruction and mesenteric lymphadenopathy were found in abdominal computed tomography and endoscopic ultrasonography. There was also a large gastric ulcer (Forrest IIa) at the greater curvature of body of the stomach. Histopathological results of the skin and stomach were consistent with diffuse large B cell lymphoma; gastric biopsy being negative for leucocyte common antigen. The patient was considered to have disseminated DLBCL. The aim of the present case report was to present the clinical, radiological, and histological characteristics of the patient, which may aid physicians in diagnosing involvement of multiple extranodal sites in DLBCL.

## 1. Introduction

Gastrointestinal tract (GIT) is the most common extranodal site for non-Hodgkin's lymphoma (NHL), constituting about 10%–15% of all NHL. Diffuse large B cell lymphoma (DLBCL) is the most common histological subtype, representing approximately 30% of NHLs. The most common primary extranodal sites of DLBCL are the gastrointestinal tract, the head and neck, and the skin/soft tissue [1]. Here, we report a case of DLBCL presenting with both gastrointestinal and dermatological manifestations.

## 2. Case Report

**Figure 1:**
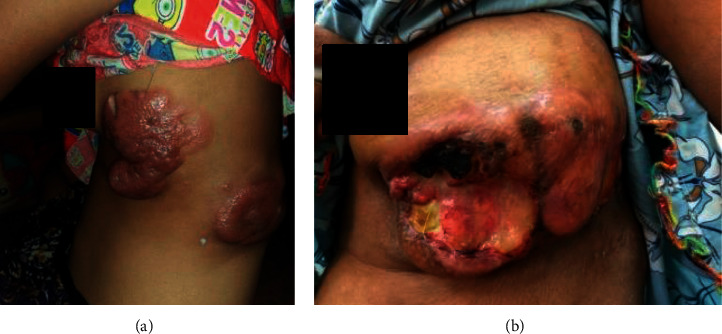
(a). Cutaneous manifestations at 2015: skin nodules on the left lower chest and loin region. (b) One of the cutaneous manifestations at the time of admission: large ulcer with some necrotic tissue (11 × 6 cm in size) at left lower chest wall.

A 39-year-old lady from a remote area was referred from dermatological unit for the large ulcerative skin lesion over the abdomen and trunk associated with progressive obstructive jaundice and significant weight loss over 1 month. Because of cholangitis with sepsis at that moment, she was decided to treat GI pathology first. On reviewing her history, since 2015, she has had progressive infiltrative skin lesions on her left lower chest (Figure 1(a)), right upper chest, and left lumber region for which indigenous medicine has been taking prior to medical consultation.

The physical examination revealed jaundice, abdominal tenderness, and multiple skin lesions on the chest and left loin region (Figure 1(b)). All the skin lesions had no bleeding or tenderness. Lymphadenopathy, organomegaly, or ascites was not detected.

The liver function test revealed high bilirubin and alkaline phosphatase. There was also hypochromic microcystic anaemia (hemoglobin, 10 g/dl; mean corpuscular volume (MCV), 76.6 fl). Lactate dehydrogenase (LDH) was 770 U/L, and tumor markers such as CA 19-9 and alpha-fetoprotein were normal. There is no abnormal finding in chest X-ray (CXR, PA view).

**Figure 2:**
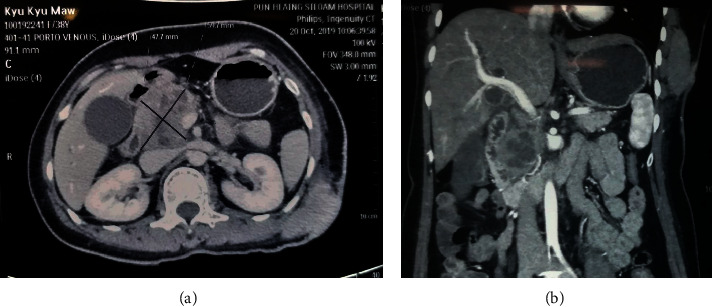
(a), (b) CT revealed malignant cystic pancreatic tumor occupying head and body with invasion to lower end of CBD and periampullary region causing biliary obstruction.

In abdominal computed tomography (CT), there was malignant cystic pancreatic tumor (6.3 × 4.7 × 3.8 cm) occupying the head and body with invasion to lower end of common bile duct (CBD) and periampullary region causing biliary obstruction and mesenteric lymphadenopathy (Figures 2(a) and 2(b)). There was no abnormal finding in the liver and spleen.

**Figure 3:**
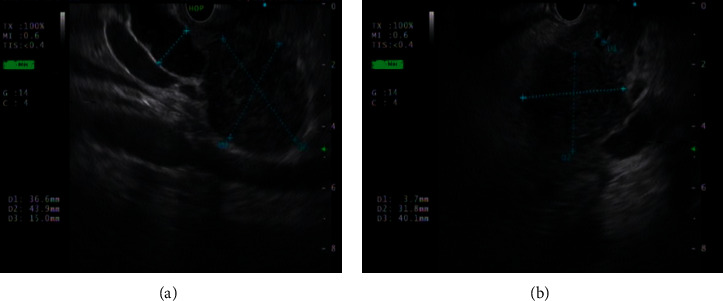
(a), (b) EUS showing a large inhomogenous hypoechoic mass (4 × 3.6 cm in diameter) in pancreatic head region with dilatation of CBD (1.4 cm) and both IHDs. Main pancreatic duct (MPD) is mildly dilated (3.4 mm) in head region.

Endoscopic ultrasound (EUS) showed a large inhomogenous hypoechoic mass (4 × 3.6 cm in diameter) in pancreatic head region with dilatation of proximal CBD and both intrahepatic ducts (IHD) (Figures 3(a) and 3(b)). EUS-guided fine needle aspiration cytology (FNAC) from pancreatic head mass found large atypical cells with hypochromatic nuclei and few papillae with stratification and irregular chromatin (positive for malignancy). However, EUS-guided fine needle aspiration biopsy (FNAB) came back as the tissue was not adequate for analysis. Straight plastic biliary stent was placed for cholangitis with long CBD stricture (5.1 cm in length).

**Figure 4:**
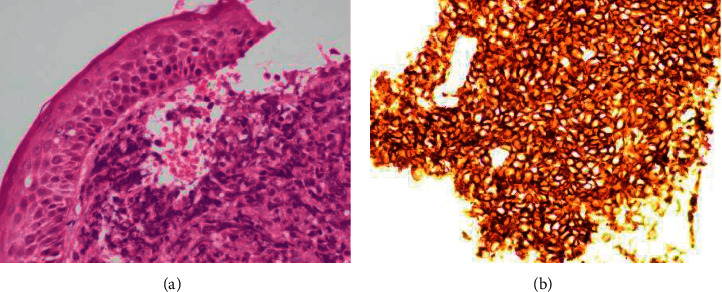
IHC analysis of skin biopsy. (a) The subcutaneous tissue infiltrated by diffuse sheet of atypical large lymphoid cells with vesicular nuclei, prominent nucleoli, and moderate cytoplasm (hematoxylin and eosin (H&E staining, x20). (b) Strongly membranous positive CD 20 in lesional lymphoid cells (IHC-CD20, x40).

On the other hand, immunohistochemical (IHC) staining of the skin biopsy revealed strongly positive CD20, high Ki67 (Figure 4), but negative expression for CD3, CD45, CD30, AE1/3, CD10, Bcl-6, Bcl-2, and MUM-1. Findings were consistent with diffuse large B cell lymphoma.

**Figure 5:**
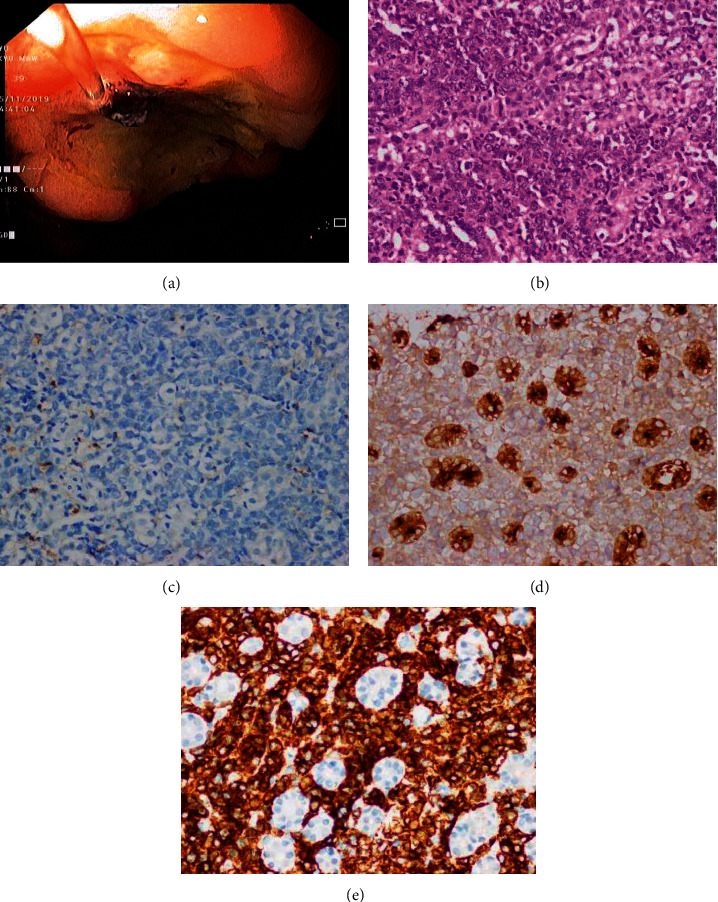
Gastroscopic biopsies and IHC staining. (a) A large gastric ulcer (Forrest IIa) at greater curvature of body of the stomach. (b) Diffuse sheets of atypical large lymphoid cells having vesicular nuclei, prominent nucleoli, and moderate eosinophilic cytoplasm. They are spilling into gastric epithelial cells (IEL, intraepithelial lymphoid cells). Mitoses are occasionally seen (H and E staining; magnification, x40). (c) LCA (leucocyte common antigen, CD 45) negative in tumor cells (IHC x40). (d) EMA (epithelial membrane antigen) negative in tumor cells and positive only in residual gastric glands (IHC x40). (e) CD 20 strongly positive in lesional large lymphoid cells and negative in gastric epithelial cells (IHC staining; magnification, x100).

On the first day of post-ERCP, the patient had hypovolaemic shock with gastrointestinal bleeding. In EGD, there was a large gastric ulcer (Forrest IIa) at the greater curvature of body of the stomach (Figure 5(a)), which would have been missed in ERCP. Combined adrenalin injection and heater probe coagulation were applied. Histopathological result of gastric ulcer initially came out as poorly differentiated adenocarcinoma or lymphoma in H&E staining (Figure 5(b)). Histochemical staining of both leucocyte common antigen (LCA) and epithelial membrane antigen (EMA) were negative in this case (Figures 5(c) and 5(d)). However, due to different anatomical sites involvement and getting the IHC result of skin biopsy just before it, the suspection of lymphoma behoved us to exclude the possibility of LCA-negative lymphoma. The subsequent IHC panel revealed diffusely positive for CD20 in these atypical cells (Figure 5(e)).

After stabilizing the general condition of the patient for a few weeks, second EUS procedure was repeated with the intention to take adequate FNA biopsy from pancreatic mass. At that time, because there was extrinsic compression of the first part of duodenum and an infiltrative lesion along second part of the duodenum, the EUS linear scope was not able to pass through luminal narrowing. Because of the cost issue, we did not proceed to IHC stainings on duodenal tissue but assumed to be the same pathology.

The final diagnosis was disseminated diffuse large B cell lymphoma (DLBCL) with cutaneous and gastrointestinal involvement. Unfortunately, the patient expired just before receiving chemotherapy in the oncology ward.

## 3. Discussion

Most DLBCLs originate in lymph nodes, but ≤40% initially present in extranodal sites [2]. Like most other NHLs, there is a male predominance with approximately 55% of cases occurring in men [3]. Sixty percentage of patients will present with advanced stage DLBCL (usually stage III or IV disease), while 40% have a more localized disease [4, 5]. The patient in this report had cutaneous manifestation since 5 years ago. At the time of admission, she presented with obstructive jaundice due to CBD stricture with pancreatic involvement and gastrointestinal bleeding due to gastric involvement.

Depending on the site of involvement, there are 2 distinct subsets: primary cutaneous DLBCL which initially presents on the skin and DLBCL accompanied by secondary spread to the skin [6]. The presence of multiple skin lesions and time of evolution at presentation were associated with poorer prognosis in secondary cutaneous DLBCL [7]. In this patient, we assumed that her cutaneous manifestation was initial presentation of disseminated DLBCL. Because of delay in seeking medical advice, we could not exclude whether gastrointestinal involvement preceded subcutaneous nodules appearance or not.

Gastrointestinal bleeding is the presenting symptom in 20–30% of gastric DLBCL cases. Lymphoma can be recognized as ulceration, diffuse infiltration, or a polypoid mass during an endoscopic examination. *Helicobacter pylori* association can be found in 63% of high-grade lymphoma and 88% of low-grade lymphoma [8]. Endoscopy finding of our patient revealed a large ulcer (Forrest IIa) at the greater curvature of body of stomach.

In pancreatic lymphoma, abdominal pain is the most common presenting symptom (83%), followed by abdominal mass (58%), weight loss (50%), jaundice (37%), acute pancreatitis (12%), small bowel obstruction (12%), and diarrhea (12%) [9]. Our patient presented with biliary obstruction due to pancreatic lesion. Theoretically, obstructive jaundice is less frequent than in pancreatic adenocarcinoma [9].

To differentiate pancreatic lymphoma from adenocarcinoma in imagings, Merkle et al. reported that findings of a bulky localized tumor in the pancreatic head without significant dilatation of the main pancreatic duct, invasive tumor growth not respecting anatomic boundaries, and the absence of calcifications and necrosis within the tumor mass, all seen more commonly in lymphoma [9, 10]. Enlarged lymph nodes below the renal vein also rules out pancreatic cancer and favors a diagnosis of pancreatic lymphoma [11]. Ultrasound or CT-guided fine needle biopsy of the pancreatic mass can also help to distinguish pancreatic lymphoma from pancreatic adenocarcinoma [12]. Unfortunately, in this patient, the tissue of FNB was not adequate for interpretation.

Staining for pan-B cell markers such as CD20 and CD79a should be performed for the diagnosis, and a much broader set of stains may be needed in cases with atypical morphological features [13]. The distinction between poorly differentiated adenocarcinoma and gastric lymphoma in endoscopic biopsies is sometimes difficult owing to the morphologic similarities that these neoplasias can share and the small amount of tissue obtained by this technique. An additional factor that may contribute to this confusion is the presence of artifactual lymphocytes resembling signet-ring cells [14]. Therefore, Arista-Nasr et al. suggested that the diagnosis of gastric lymphoma must be excluded using histochemical and immunohistochemical studies if a poorly differentiated neoplasm consistent with signet-ring adenocarcinoma is found in an endoscopic biopsy with artificial changes [14].

In our patient, IHC staining of gastric biopsy was LCA-negative, CD 20-positive DLBCL. Rare cases of LCA-negative large B cell lymphomas previously reported have an extranodal presentation, urinary bladder [15], thyroid gland [16], and cerebellum [17], and have been reclassified as plasmablastic lymphoma or ALK-positive large B cell lymphoma in literature. Loss of LCA expression on lymphoma cells usually suggests that neoplastic transformation has occurred during the differentiation pathway to plasma cells just before its CD20 expression [16].

In conclusion, we reported a rare case of diffuse large B cell lymphoma with cutaneous and gastrointestinal involvement. Pancreatic head mass was also assumed to be lymphoma, although we could not confirm the diagnosis. Histopathological diagnosis with histochemical and IHC studies are important in distinguishing lymphoma from adenocarcinoma. Although most cases of DLBCL respond well to chemotherapy, our patient could not undergo adequate treatment for her disease because of poor performance status and large burden of disease since the time of presentation, leading to her death.
